# Re-evaluating early high-frequency oscillatory ventilation in moderate-to-severe pediatric ARDS: evidence from a genetic matching analysis

**DOI:** 10.3389/fmed.2026.1725518

**Published:** 2026-03-25

**Authors:** Feifan Jin, Ziqing Quan, Hongxing Dang

**Affiliations:** Department of PICU Children’s Hospital of Chongqing Medical University, National Clinical Research Center for Child Health and Disorders, Ministry of Education Key Laboratory of Child Development and Disorders, Chongqing Key Laboratory of Pediatrics, China International Science and Technology Cooperation Base of Child Development and Critical Disorders, Chongqing, China

**Keywords:** genetic matching, high-frequency oscillatory ventilation, intensive care, mechanical ventilation, pediatric acute respiratory distress syndrome

## Abstract

**Background and aims:**

Pediatric acute respiratory distress syndrome (PARDS) carries high mortality in pediatric intensive care units (PICUs). The clinical benefit of early high-frequency oscillatory ventilation (HFOV) for moderate-to-severe PARDS remains controversial. This study aimed to compare clinical outcomes between HFOV and conventional mechanical ventilation (CMV) using a robust genetic matching approach.

**Methods:**

In this retrospective case–control study, children with moderate-to-severe PARDS admitted to the two PICUs of Chongqing Medical University from January 2012 to June 2024 were analyzed in a pre-specified 7-day landmark cohort (sustained invasive MV ≥ 7 days). Genetic matching in R software was applied to balance baseline characteristics between HFOV and CMV groups. The primary outcome was 28-day mortality; secondary outcomes included ventilator-free days (VFD), PICU-free days (IFD), survival time, and survival rates stratified by pre-intubation PaO_2_/FiO_2_ (P/F) ratios.

**Results:**

After matching, 53 patients were included in each group with well-balanced baseline variables. Sensitivity (Rosenbaum bounds) and robustness (nearest-neighbor matching) analyses confirmed the stability of the matched results. The 28-day mortality was significantly higher in the HFOV group than in the CMV group (49.1% vs. 28.3%, *p* = 0.04), whereas VFD and IFD did not differ significantly. Logistic regression indicated that HFOV was independently associated with higher 28-day mortality [adjusted OR = 2.47 (95% CI 1.02–5.98), *p* = 0.04]. In stratified analysis, the 28-day survival rate for moderate PARDS (200 ≥ P/*F* > 100) was markedly lower with HFOV than CMV (0.41 vs. 0.82, *p* = 0.01).

**Conclusion:**

In this 7-day landmark cohort, early HFOV may not provide extra benefit in moderate-to-severe PARDS and might be associated with higher mortality, whereas CMV was linked with lower observed 28-day mortality. Further prospective studies are warranted.

## 1 Introduction

Acute respiratory distress syndrome (ARDS) is a common, high-mortality lung condition in intensive care units (ICU) (1). Despite lung-protective conventional mechanical ventilation (CMV), mortality and practice variation remain substantial in moderate-to-severe ARDS. High-frequency oscillatory ventilation (HFOV) is a lung-protective mode of ventilation. It uses high respiratory rates along with continuous mean airway pressure (MAP) and very low tidal volumes. This approach helps prevent alveolar collapse and may reopen collapsed alveoli (2, 3). It also reduces peak airway pressure to prevent lung over distension and injury. By improving gas exchange more evenly and effectively, HFOV is thought to enhance outcomes in ARDS (4).

HFOV has been in clinical use for several decades. Despite its theoretical advantages (3, 5, 6), studies in adult ARDS have found that early application of HFOV does not significantly reduce mortality and may even increase in-hospital mortality (7). In the treatment of pediatric ARDS (PARDS), HFOV is also widely used, but its impact on clinical outcomes in children with moderate to severe ARDS remains unclear. Some studies suggest that HFOV may offer short-term improvements in oxygenation for PARDS, but it has also been associated with higher mortality risk (8, 9), however, this association remains controversial (10–12). Due to the lack of consistent usage standards and unified clinical management protocols, as well as the substantial heterogeneity of existing studies, the comparison of HFOV and CMV in treating moderate-to-severe PARDS remains insufficiently studied. Notably, these prior cohorts included a considerable proportion of patients with mild PARDS, further limiting inferences for moderate-to-severe disease.

Given the significant differences in pathological characteristics, treatment approaches, and prognosis between children with moderate to severe ARDS and those with mild ARDS (13), and the lack of well-controlled studies and in-depth analysis of HFOV in moderate to severe PARDS, current evidence is insufficient to determine its association with clinical outcomes. The latest 2023 PARDS expert consensus also does not provide a clear recommendation on whether HFOV can replace CMV (14).

Therefore, we conducted a rigorous case–control study using a 1:1 genetic matching algorithm to analyze children with moderate to severe ARDS who received HFOV or CMV within the first 7 days after the diagnosis of moderate-to-severe PARDS. By comparing outcomes such as 28-day mortality, ventilator-free days (VFD), and PICU-free days (IFD), we assessed whether the early HFOV, compared with CMV, was associated with differences in clinical outcomes of these patients. Through this study, we aim to provide more concrete evidence to support decision-making regarding the necessity of early HFOV application in moderate to severe PARDS.

## 2 Materials and methods

This study is a retrospective case–control study reported in accordance with the Strengthening the Reporting of Observational Studies in Epidemiology (STROBE) guidelines (15). The study population consisted of children with moderate to severe PARDS admitted to two PICUs at the Children’s Hospital of Chongqing Medical University in China between January 2012 and June 2024. The study was conducted in accordance with the Declaration of Helsinki and approved by the Ethics Committee of Children’s Hospital of Chongqing Medical University (Approval No. 145/2024; approval date: April 15, 2024). Given the retrospective nature of the study and the use of anonymized data, the requirement for informed consent was waived by the ethics committee.

### 2.1 Inclusion and exclusion criteria

*Inclusion criteria*: (1) Patients who were primarily diagnosed with moderate to severe PARDS within 48 h of PICU admission and received invasive mechanical ventilation; (2) Continuous HFOV and/or CMV treatment within the first 7 days after diagnosis; (3) Age between 29 days (corrected gestational age) and 18 years.

*Exclusion criteria*: (1) Patients who received invasive mechanical ventilation before PICU admission or prior to the diagnosis of moderate to severe ARDS; (2) Patients dependent on mechanical ventilation due to underlying diseases such as chronic neuromuscular, respiratory, genetic metabolic diseases, malignancies, or cardiovascular conditions; (3) Patients with end-stage chronic disease or organ failure, where the poor prognosis is not primarily due to ARDS; (4) Patients receiving extracorporeal membrane oxygenation (ECMO) treatment before PARDS diagnosis; (5) Patients with a PICU stay or mechanical ventilation duration of less than 7 days, to define a 7-day landmark (sustained-ventilation) cohort.

### 2.2 Definitions and outcome measures

The diagnosis of moderate to severe ARDS in this study was determined by retrospectively applying the Pediatric Acute Lung Injury Consensus Conference (PALICC) criteria (14). The “HFOV group” was defined as patients who received HFOV treatment for more than 24 h within the first 7 days after the diagnosis of moderate to severe PARDS, while the “CMV group” was defined as those who were treated exclusively with CMV during the first 7 days. To characterize time-varying exposure within this 7-day window, we extracted daily ventilator-mode data and summarized the total number of days on CMV and on HFOV within days 1–7 after diagnosis (a fixed 7-day exposure window). Accordingly, the estimand of this study is the association between a 7-day HFOV exposure pathway versus a 7-day CMV-only pathway and 28-day outcomes among children requiring sustained invasive ventilation for ≥7 days.

Due to the absence of a standardized treatment protocol, the initiation, optimization, and discontinuation of HFOV and CMV were at the discretion of the attending physicians in each PICU. We followed a lung-protective ventilation strategy (16), maintaining SpO_2_ between 88% and 95%, and allowing a certain degree of permissive hypercapnia and hypoxemia (PaCO_2_ 50–70 mmHg, PaO_2_ 55–60 mmHg), depending on the patient’s tolerance.

The primary outcome was 28-day mortality in the 7-day landmark cohort. Secondary outcomes included VFD and IFD within 28 days. VFD was defined as the number of days the patient survived without mechanical ventilation within the 28-day period. For instance, if a patient received mechanical ventilation from Day 1 to Day 7 after the diagnosis and survived without requiring further ventilation until Day 28, the VFD would be 21 days. If the patient died within the 28 days, the VFD was automatically recorded as 0, regardless of the number of days spent on mechanical ventilation. IFD was defined as the number of days the patient survived outside of the PICU within the 28-day period. For example, if a patient was discharged from the PICU on Day 10 and survived for the remaining 18 days without being readmitted to the PICU, the IFD would be 18 days. If the patient died or remained in the PICU within 28 days, the IFD would automatically be 0. This method ensures that the impact of death does not confound the evaluation of ventilation and PICU stay durations.

### 2.3 Data collection

Data were collected from electronic medical records and follow-up data. The following information was obtained: general demographic characteristics (age, sex, weight), comorbidities (including neurological, cardiovascular, respiratory, renal, gastrointestinal, hematologic, immunologic, metabolic, congenital and genetic diseases, and malignancies), and severity scores for moderate to severe ARDS (Pediatric Index of Mortality 3 [PIM3] at the diagnosis of moderate-to-severe PARDS and the highest Pediatric Logistic Organ Dysfunction [PELOD] score recorded during the first 7 days).

Additionally, data were collected on PaO_2_/FiO_2_(P/F) ratio before intubation, whether PARDS were triggered by lower respiratory tract infections (LRTI), the presence of positive microbiological examinations (blood and/or sputum), and treatment information(daily ventilator use and type during the first 7 days and main ventilator parameters, and the use of *β*-agonists, corticosteroids, diuretics, surfactant, packed red blood cells (PRBC) transfusion, ECMO, re-intubation, prone positioning, neuromuscular blockers and duration of moderate to deep sedation). The conditions of multiple organ dysfunction syndrome (MODS) prior to ventilation were also recorded. Outcome data included 28-day mortality, VFD and IFD.

### 2.4 Statistical analysis

The study used R version 4.4.1 for genetic matching and analysis of data pre- and post-Genetic Matching. Continuous variables were described as mean ± standard deviation (x̄ ± s) or median (Q1, Q3), while categorical variables were expressed as frequency (*n*) and percentage (%). Group differences for continuous variables were analyzed using independent sample *t*-tests or Mann–Whitney *U* test (*U*-tests), and chi-square tests were applied for categorical variables. All statistical tests were two-sided, and a *p* value <0.05 was considered statistically significant. Missingness was minimal for variables used in matching and outcome analyses; therefore, analyses were performed on available data without imputation.

Logistic regression was used to evaluate the association between HFOV and 28-day mortality, and the Hosmer–Lemeshow test was applied to assess the model’s fit. A generalized linear model was used to evaluate the association between HFOV on secondary outcomes, reporting regression coefficients, standard errors, and the confidence intervals (95% CI). Kaplan–Meier survival curves were compared using the log-rank test. Subgroup analyses based on P/F stratification were pre-specified.

Based on a literature review and expert opinions (14, 17), the covariates used for matching included age, weight, whether lower respiratory tract infections triggered PARDS, the presence of comorbidities, MODS, PIM3 score, and P/F ratio before intubation, as well as the use of corticosteroids, duration of neuromuscular blocker use, prone positioning, and total duration of moderate to deep sedation during the first 7 days of ventilation. The same pre-specified covariate set was used in both the pre- and post-matching regression models. For post-matching regression analyses, robust variance estimation clustered by matched pair was used to account for the 1:1 matched design.

After cleaning the data and categorizing variables, 1:1 genetic matching was performed using the GenMatch function in R. Genetic matching is an optimization-based method that employs genetic algorithms to identify individuals in the HFOV and CMV groups who are most similar across multiple covariates (18). After matching, standardized mean differences (SMD) were used to assess covariate balance between the HFOV and CMV groups, with an absolute SMD value below 0.1 considered indicative of good balance. Sensitivity analysis was conducted using the Rosenbaum Bounds method, and robustness was evaluated through nearest-neighbor matching. The genetic matching procedure and the overall statistical analysis plan were reviewed by a biostatistician.

## 3 Results

### 3.1 General data

**Figure 1:**
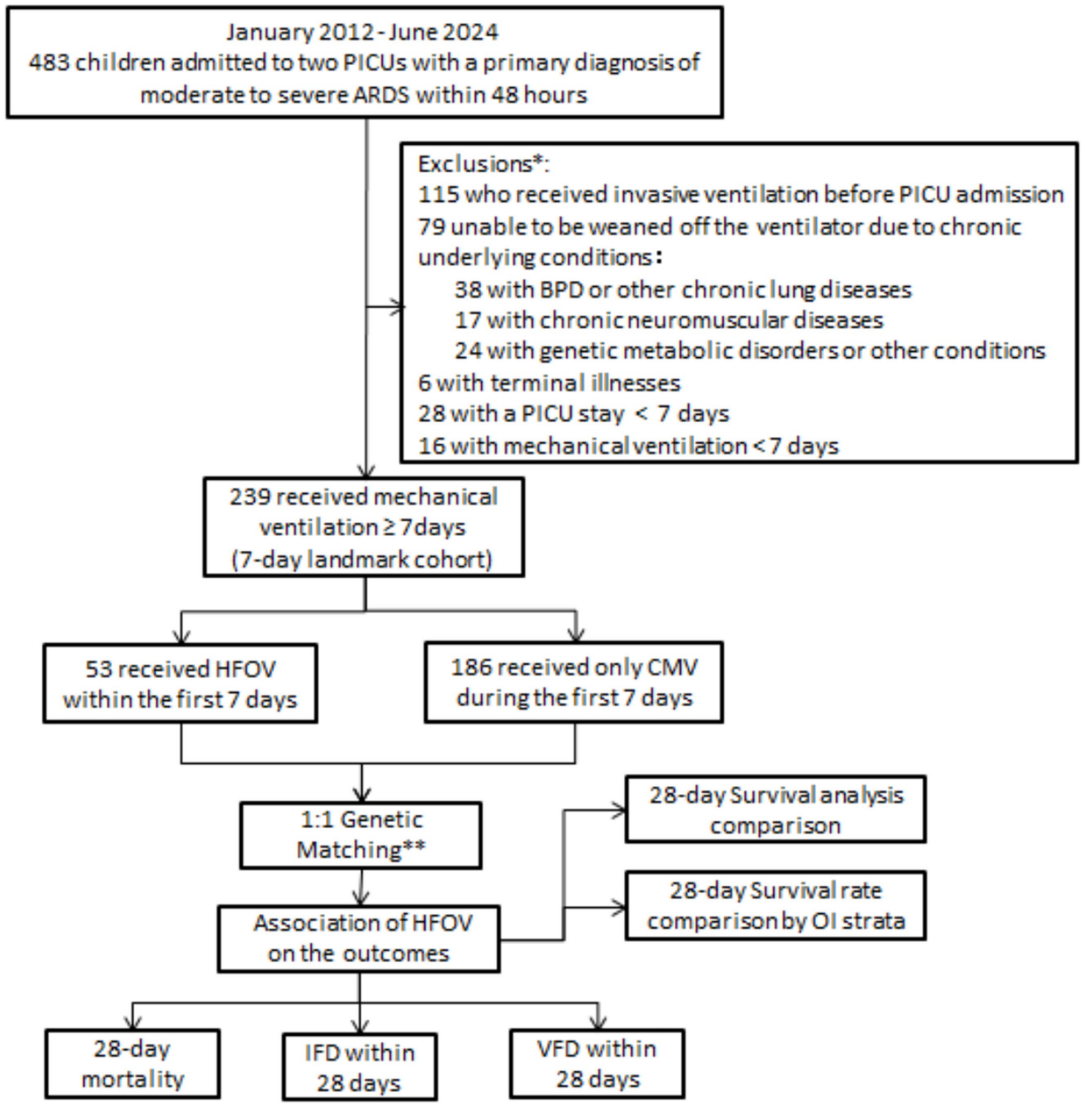
Research flow chart. *Counts are mutually exclusive; **Genetic matching was performed using the GenMatch function in R.

During the study period, a total of 483 children with moderate to severe ARDS were admitted to the PICUs. Of these, 239 patients met the study’s inclusion and exclusion criteria. As shown in Figure 1 1:1 genetic matching was applied to evaluate the impact of HFOV on outcomes.

In the HFOV group (*n* = 53), the number of CMV days within the first 7 days was median 3 (IQR 1–5), and the number of HFOV days was median 4 (IQR 2–6). Initiation timing categories within the first 7 days and HFOV duration distributions are summarized in Supplementary Table S1.

### 3.2 Comparison of baseline characteristics of PARDS between the two groups pre- and post-genetic matching

**Figure 2:**
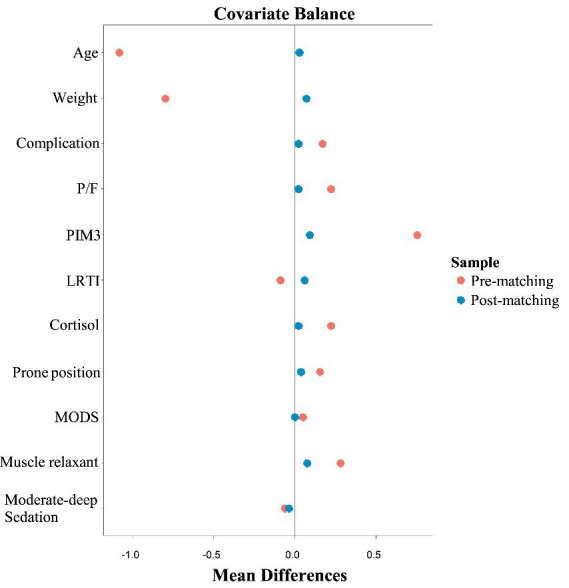
Covariate balance plot before and after genetic matching. |SMD| < 0.1.

After genetic matching, all cases in the HFOV group were successfully matched with corresponding cases in the CMV group, resulting in 53 cases in each group. This created a well-balanced study cohort without duplication. The SMD for all covariates included in the matching were less than 0.1 (Figure 2).

Before matching, there were significant differences between the HFOV and CMV groups in several baseline characteristics, including weight, PIM3 score, comorbidities, P/F ratio, and the use of corticosteroids and prone positioning during the first 7 days of ventilation (*p* < 0.05). After genetic matching, there were no statistically significant differences between the two groups in any of the baseline characteristics (*p* > 0.05) (Table 1).

**Table 1:** Characteristics of PARDS pre- and post-genetic matching.

Variable	HFOV (*n* = 53)	CMV (*n* = 186) pre-matching	Statistic (u/x)	*p*	CMV (*n* = 53) post-matching	Statistic (u/x)	*p*
Female [*n*(%)]	29 (54.7%)	103 (55.4%)	0	>0.99	31 (58.5%)	0.038	0.84
Age, months (M[IQR])	17 (6,28)	17 (7, 71)	5,645	0.11	12 (5, 30)	1283.5	0.44
Weight, kg (M[IQR])	8.5(6.5,12.0)	10 (6.5, 18.5)	5,866	0.04*	8.5 (5.2, 11.5)	1335.5	0.66
PIM3 (M[IQR])	0.8(0.7,0.9)	0.61(0.1,0.9)	3181.5	<0.001	0.76 (0.62, 0.91)	1249.5	0.32
PELOD (M[IQR])	6.0(4.0,10.0)	5.5(2.0,10.0)	4505.5	0.34	7 (5, 10)	1,562	0.31
Comorbidities [*n*(%)]	44(83.0%)	123(66.1%)	4.8	0.03*	44 (83.0%)	0	>0.99
LRT risk factor [*n*(%)]	34(64.2%)	136(73.1%)	1.2	0.27	30 (56.6%)	0.355	0.55
Blood culture+ [n(%)]	8(15.1%)	25(13.4%)	0	0.94	9 (17.1%)	0	>0.99
Sputum culture+ [*n*(%)]	21(39.6%)	65(34.9%)	0.2	0.64	25 (47.2%)	0.346	0.56
P/F (M[IQR])	66.5(47.7, 123.1)	108.7 (66.1, 165.7)	6422.5	0.001*	72.4 (53.2, 123.1)	1,534	0.42
P/*F* < 100 [*n*(%)]	38 (71.7%)	92 (49.5%)	7.35	0.007*	37 (69.81%)	0	>0.99
ICU reintubation [*n*(%)]	5 (9.4%)	12 (6.5%)	Fisher	0.54	3 (5.7%)	Fisher	0.72
Corticosteroid [*n*(%)]	32(60.4%)	71(38.2%)	7.4	0.006*	32 (60.4%)	0	>0.99
Prone positioning [*n*(%)]	15(28.3%)	24(12.9%)	6.1	0.01*	12 (22.6%)	0.199	0.66
Medium deep sedation, days (M[IQR])	10 (8,15)	11 (7,16)	5,097	0.70	12 (7, 16)	1,463	0.71
NMB [*n*(%)]	46 (86.8%)	164 (88.2%)	0	0.97	51 (96.2%)	Fisher	0.16
NMB, days (M[IQR])	6 (2,12)	4 (2,8)	4,106	0.06	5 (2, 10)	1331.5	0.65
Diuretic [*n*(%)]	46 (86.8%)	139 (74.7%)	2.8	0.09	48 (90.6%)	0.094	0.76
CRRT [*n*(%)]	7 (13.2%)	13 (7.0%)	Fisher	0.16	2 (3.8%)	Fisher	0.16
Surfactant [*n*(%)]	5 (9.4%)	6 (3.2%)	Fisher	0.07	3 (5.7%)	Fisher	0.72
RBC transfusion [*n*(%)]	30 (56.6%)	104 (56.0%)	0	>0.99	27 (50.9%)	0.152	0.69
ECMO [*n*(%)]	2 (3.8%)	2 (3.8%)	Fisher	>0.99	3 (5.7%)	Fisher	>0.99
MODS [*n*(%)]	24 (45.3%)	76 (40.9%)	0.175	0.676	23 (43.4%)	0	>0.99

Group differences for continuous variables were analyzed using *U*-tests, and chi-square tests or Fisher’s exact tests were applied for categorical variables. *p < 0.05.

### 3.3 Sensitivity and robustness analysis

**Figure 3:**
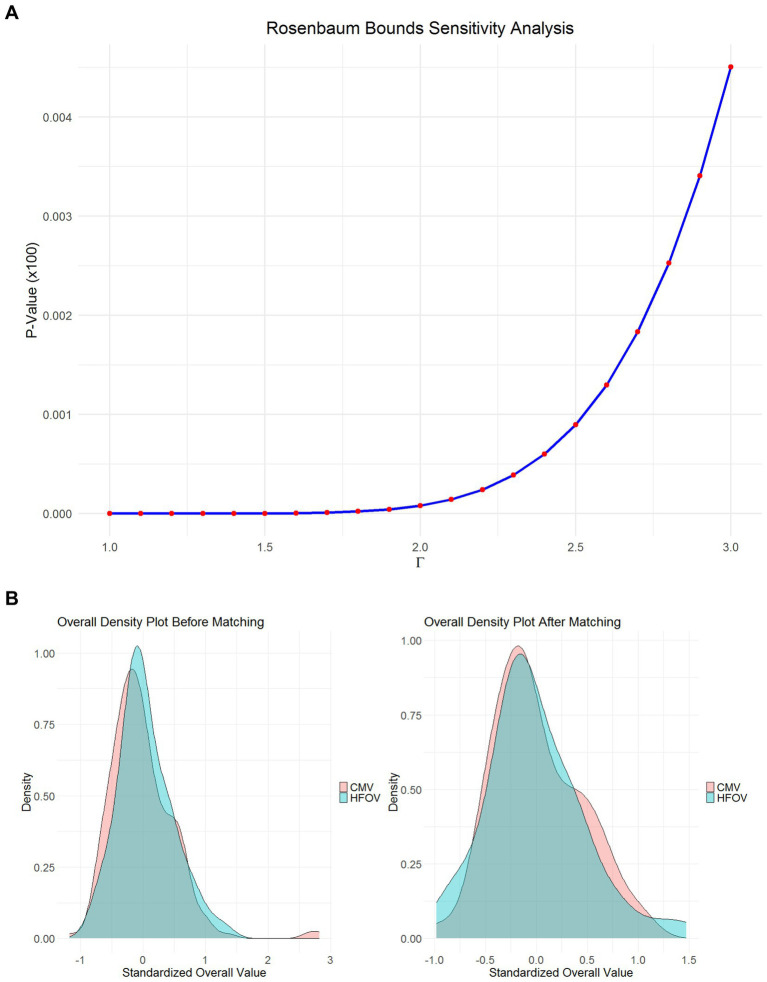
**(A)** Rosenbaum bounds sensitivity analysis plot showing the impact of unobserved confounding factors on the robustness of the study’s findings; **(B)** Overall probability density plot of standardized variables before and after genetic matching, illustrating the improvement in covariate balance between the HFOV and CMV groups.

To assess the potential impact of unobserved bias on the study results, a Rosenbaum Bounds sensitivity analysis was conducted. This analysis evaluates the extent to which unobserved confounding factors could affect the study’s conclusions. The results showed that even with a Gamma value increased to 3, the *p*-value remained very small (<0.005). This suggests that the observed association is relatively robust to moderate levels of hidden bias (Figure 3A).

To verify the robustness of the genetic matching results, we used the same covariates and applied the Nearest Neighbor Matching method for validation. Figure 3B shows the density distribution of the standardized overall values for the HFOV and CMV groups before and after Nearest Neighbor Matching.

Before matching, there was little overlap between the two groups near the standardized value of 0, with most SMDs greater than 0.1. The HFOV group exhibited a distinct peak, while the CMV group showed a more dispersed density. This indicated significant differences and inconsistencies in the variable distributions between the two groups. After matching, the distributions became more consistent, significantly reducing intergroup imbalances. This demonstrates that the balance between the groups remained good after using a different matching method, confirming the validity and stability of the genetic matching.

### 3.4 Comparison of primary and secondary outcomes between the two groups pre- and post-genetic matching

Before genetic matching, there were no statistically significant differences between the HFOV and CMV groups in terms of 28-day mortality, VFD, and IFD. After matching, the 28-day mortality rate was significantly higher in the HFOV group compared to the CMV group (*p* = 0.04), but the impact on other secondary outcomes (VFD and IFD) remained statistically non-significant (*p* > 0.05) (Table 2).

**Table 2:** Comparison of outcomes between the HFOV and CMV groups pre- and post-genetic matching.

Variables	HFOV (*n* = 53)	CMV (*n* = 186) pre-matching	Statistic (u/x)	*p*	CMV (*n* = 53) post-matching	Statistic (u/x)	*p*
28-day mortality [*n* (%)]	26 (49.1%)	72 (38.7%)	1.423	0.23	15 (28.3%)	3.977	0.04*
IFD (M [IQR])	15 (9, 18)	14 (7, 19)	4,941	0.97	11 (7, 18)	1,313	0.56
VFD (M [IQR])	18 (13, 20)	17 (12, 21)	4,759	0.70	16 (12, 21)	1,342	0.69

Group differences for continuous variables were analyzed using *U*-tests, and chi-square tests were applied for categorical variables. *p < 0.05.

### 3.5 Association of HFOV with outcomes of PARDS pre- and post-genetic matching

In the pre-matching regression analysis, after adjustment for the pre-specified covariates, HFOV was not significantly associated with the primary or secondary outcomes (28-day mortality, IFD, and VFD). After genetic matching, HFOV was associated with higher 28-day mortality (adjusted OR = 2.474, 95% CI 1.024–5.979; *p* = 0.04) (Table 3). The mortality model showed acceptable calibration by the Hosmer–Lemeshow goodness-of-fit test (*p* > 0.05). Post-matching regression was performed in the matched cohort with the same pre-specified covariate set as in the pre-matching models.

**Table 3:** Regression analysis of the association of HFOV with outcomes in PARDS pre- and post-genetic matching.

Variables	Pre- or post-genetic matching	SE	Wald *χ*^2^	Effect estimate (95% CI)	*p*
28-day mortality	Pre-	0.355	1.704	1.589 (0.793, 3.183)	0.19
	Post-	0.45	4.051	2.474 (1.024, 5.979)	0.04*
IFD	Pre-	1.211	0.383	0.749 (−1.624, 3.122)	0.53
	Post-	1.506	0.625	1.191 (−1.761, 4.143)	0.43
VFD	Pre-	1.061	1.11	1.118 (−0.961, 3.197)	0.29
	Post-	1.285	0.876	1.203 (−1.316, 3.721)	0.35

Logistic regression was used for 28-day mortality (effect estimate reported as adjusted OR, 95% CI). Linear models were used for IFD and VFD (effect estimate reported as *β*, adjusted difference in days, 95% CI). SE and Wald *χ*^2^ correspond to the regression coefficient in each model. Covariates included in both pre- and post-matching models were: age, weight, LRTI-triggered PARDS, comorbidities, MODS, PIM3, pre-intubation P/F ratio, corticosteroid use, duration of neuromuscular blocker use, prone positioning, and total duration of moderate-to-deep sedation during the first 7 days. *p < 0.05.

### 3.6 Survival analysis between the HFOV and CMV groups before and after genetic matching

**Figure 4:**
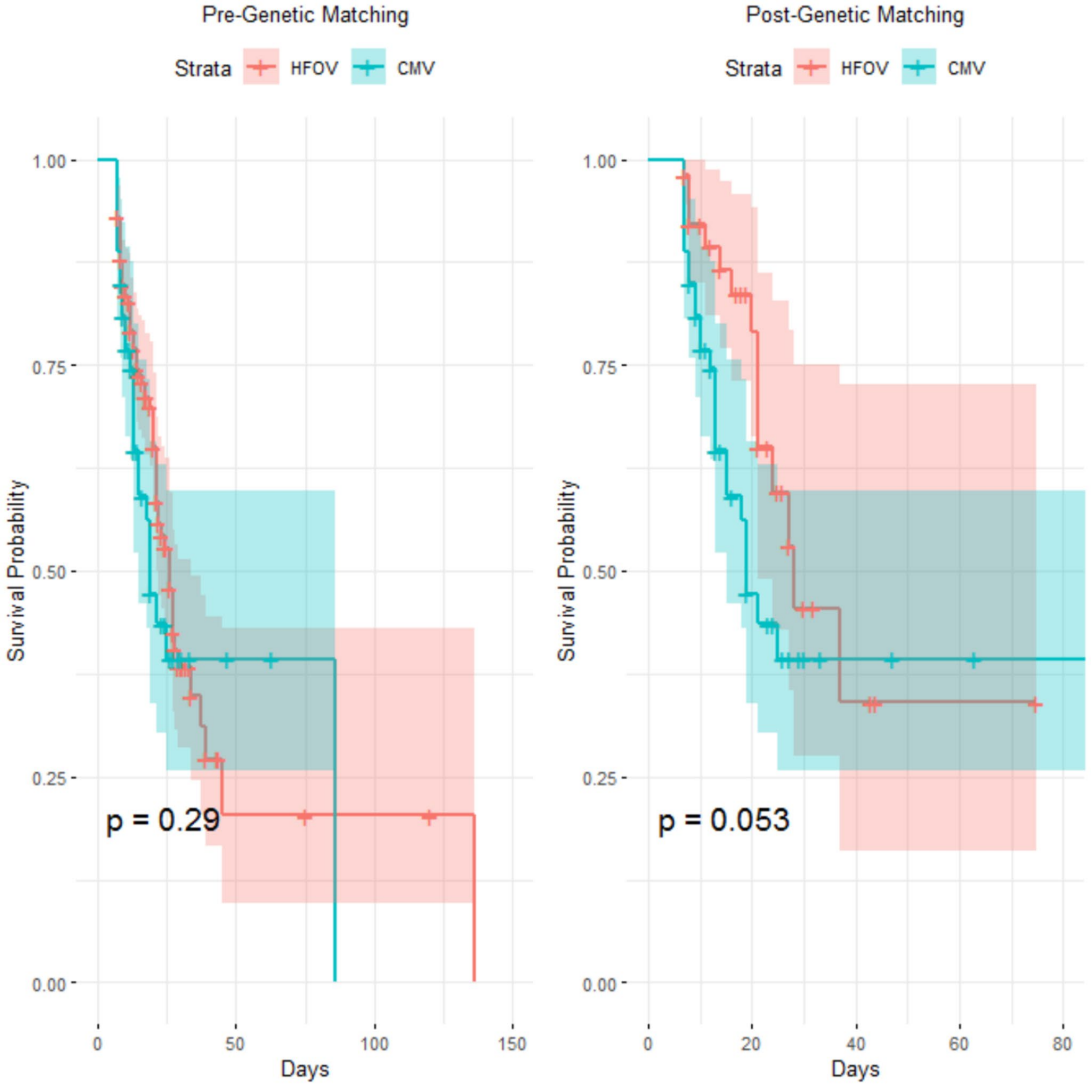
Survival analysis of PARDS treated with HFOV vs. CMV. Kaplan–Meier survival curves comparing the survival times of children treated with HFOV versus CMV before and after genetic matching.

Kaplan–Meier survival curves were used to compare survival times between the HFOV and CMV groups before and after genetic matching. The results showed that, although the survival curves of the two groups diverged within the first 20 days after matching, the Log-rank test indicated that the difference in survival between the groups was not statistically significant (*p* = 0.053) (Figure 4).

### 3.7 Association of HFOV and CMV on patient survival rates under different P/F after genetic matching

**Figure 5:**
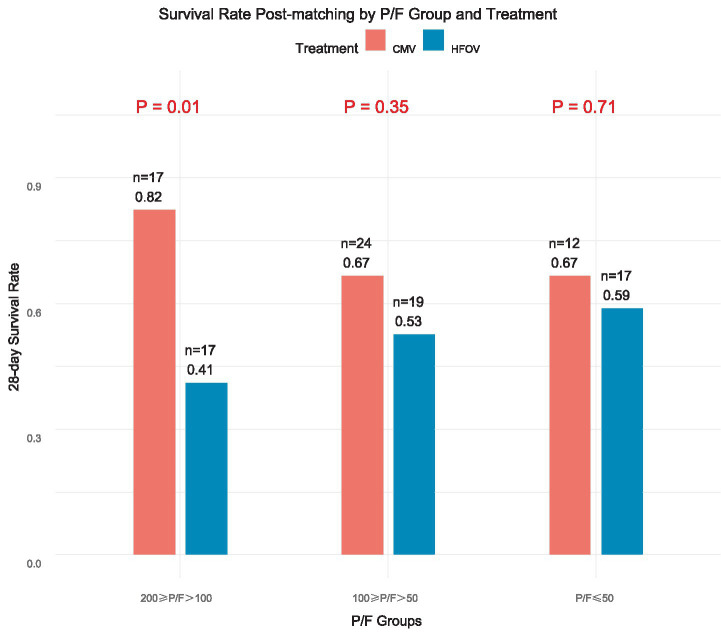
Comparison of the impact of HFOV and CMV on patient survival rates in different P/F subgroups post-genetic matching. Comparison of 28-day survival rates in different PaO_2_/FiO_2_ (P/F) ratio subgroups (200 ≥ P/F > 100, 100 ≥ P/F > 50, and P/F ≤ 50) between the HFOV and CMV groups after genetic matching.

In the moderate PARDS group (200 ≥ P/F > 100), the 28-day survival rate in the HFOV group was significantly lower than in the CMV group (*p* = 0.01). However, in the more severe PARDS groups (100 ≥ P/F > 50 and P/*F* ≤ 50), the survival rate gap between HFOV and CMV gradually narrowed, with patients treated with CMV generally having higher survival rates than those treated with HFOV, although the differences were not statistically significant. Figure 5 shows the comparison of 28-day survival rates between the HFOV and CMV groups across different P/F strata after genetic matching.

## 4 Discussion

This study used genetic matching to evaluate the impact of early (within 7 days) HFOV on outcomes in children with moderate to severe ARDS. Compared with CMV, HFOV was associated with higher 28-day mortality in children with moderate-to-severe ARDS. However, HFOV showed no significant association with secondary outcomes such as VFD and IFD within 28 days. This discrepancy may be attributed to the different nature of primary and secondary outcomes. This finding indicates that HFOV may not meet expectations in the treatment of moderate to severe PARDS, particularly showing a more pronounced negative impact on survival outcomes in moderate PARDS. These findings raise important considerations regarding the clinical application of HFOV in pediatric patients.

A notable strength of this study is the use of high-quality genetic matching. Due to the extremely strict inclusion and exclusion criteria set before the study, we successfully performed 1:1 non-redundant genetic matching between the HFOV and CMV groups. This highly matched design significantly reduced the potential impact of confounding bias on the study results, thereby enhancing the credibility of the conclusions. Additionally, sensitivity and robustness analyses further validated the effectiveness and reliability of the matching process. To our knowledge, this is among the first pediatric comparative studies to use genetic matching in moderate-to-severe PARDS.

Before matching, there were no significant differences in primary and secondary outcomes between the groups. However, after matching, the results showed that the 28-day mortality in the HFOV group was significantly higher than in the CMV group. Further regression analysis revealed that HFOV was associated with a lower 28-day survival proportion in PARDS. Nevertheless, both before and after matching, HFOV had no statistically significant effect with secondary outcomes (IFD and VFD). Moreover, HFOV did not show a significant impact on survival time in PARDS, either before or after matching, which suggests that the primary determinants of patient survival in moderate to severe ARDS are likely multi-dimensional. The discrepancy in the effects of HFOV on primary and secondary outcomes may be attributed to the distinct characteristics of these outcomes.

Consistent with findings from some previous studies on adult ARDS, research has shown that while HFOV may improve oxygenation in the short term, it does not significantly enhance long-term survival rates and may even be harmful in certain cases (7, 19). In our study, even after adjusting for multiple covariates post-matching, HFOV still demonstrated a higher 28-day mortality rate, suggesting that it may not be suitable for children with moderate to severe PARDS. This aligns with the findings of Maitra et al. (20), whose meta-analysis of adult ARDS patients concluded that HFOV should not be used as a routine treatment. Additionally, some studies do not support the use of HFOV as a “last-resort” measure for patients with respiratory failure (21). The reasons for these outcomes may be related to the mechanical ventilation characteristics of HFOV. In ARDS, lung compliance and oxygenation capacity are significantly reduced, although HFOV can maintain low tidal volume ventilation, but it may lead to hemodynamic instability and even affect perfusion pressure in the heart and brain, especially in cases of moderate ARDS where hypoxemia has not yet caused circulatory dysfunction, in this condition, HFOV could potentially accelerate this process. Furthermore, HFOV may also contribute to ventilator-associated lung injury, especially in children with heterogeneous lung compliance. Excessive mean airway pressure can lead to regional overdistension and insufficient recruitment of collapsed alveoli, resulting in volutrauma and atelectrauma. These mechanical stresses may further impair gas exchange, exacerbate lung injury, and contribute to the increased risk of mortality observed in our study.

Unlike adult studies, the pediatric field lacks high-quality controlled studies on HFOV. Some existing studies suggest that HFOV may help improve outcomes in pediatric patients (22–25), or that its effects are similar to other ventilation methods (26, 27), while others indicate that HFOV does not improve outcomes and may even lead to adverse effects (9). These markedly divergent findings may indicate that the effectiveness of HFOV differs across age groups, underlying pathophysiology, or specific ARDS phenotypes (13). Therefore, through rigorous case matching and multivariable adjustments, our study provides a more comprehensive and accurate assessment of the effects of HFOV in moderate to severe PARDS. Our findings further support the association between HFOV and increased short-term mortality in children with moderate to severe ARDS.

This study did not find significant differences in IFD and VFD between the HFOV and CMV groups. This suggests that the impact of HFOV on patient outcomes is multidimensional. Although HFOV may be associated with higher mortality risk in certain patients, it might still provide oxygenation benefits in select PARDS subgroups, potentially prolonging dependence on mechanical ventilation or hospital stay (28). Additionally, survival time is influenced by multiple factors, and a comprehensive analysis requires consideration of the patient’s clinical background, follow-up data, and the specific distribution of death times. Moreover, although baseline characteristics were well matched, the intrinsic variability in ventilator settings, such as higher mean airway pressure (mPaw) and pressure amplitude (ΔP) in HFOV or relatively higher tidal volumes in CMV, may also contribute to differences in physiological responses and clinical outcomes.

Stratified analysis based on P/F ratios revealed heterogeneous effects of HFOV across severity subgroups across varying severities of PARDS. In patients with moderate PARDS (200 ≥ P/*F* > 100), 28-day survival was higher in the CMV group than in the HFOV group, indicating an unfavorable observed association between early HFOV and survival in moderate PARDS. However, in patients with more severe PARDS (100 ≥ P/*F* > 50 and P/*F* ≤ 50), although the survival gap narrowed, HFOV still did not demonstrate survival advantage. Overall, patients treated with CMV had a higher survival rate than those treated with HFOV.

This finding has significant clinical implications, indicating that the use of HFOV in children with moderate PARDS should be approached with caution. For patients with less severe conditions, CMV may provide better survival rates and treatment outcomes. However, this does not exclude the potential benefits of HFOV in certain subgroups, such as patients with severe hypoxemia who do not respond well to conventional ventilation. In the early stages of HFOV use, it may temporarily help improve PaO2 levels (22), as some studies have suggested that the reduction in PARDS mortality may be primarily related to oxygenation improvement rather than improvements in respiratory mechanics (29).

The results of this study suggest that HFOV may carry a potential risk of increasing mortality in certain cases, thus patient selection should be approached with greater caution. In clinical practice, the use of HFOV should be carefully weighed against the individual patient’s condition and risks, and its effects should be evaluated in combination with other treatment modalities. Clinicians should utilize the input of a multidisciplinary team and tailor the treatment plan to the specific needs of each patient to ensure the best possible outcomes.

However, this study has several limitations. First, the inherent selection bias and information bias of a retrospective design may have influenced the results. Although strict inclusion criteria and refined genetic matching improved baseline balance between groups, these biases, including possible era-related confounding over the long enrollment period (2012–2024) and the 7-day landmark restriction excluding early deaths, cannot be completely eliminated. Second, the study was conducted at two independent PICUs within the same institution of southwestern China, which may limit the generalizability of the findings. Clinical practices, monitoring intensity, and healthcare resource availability in these PICUs may differ from those in other countries or multicenter networks. Moreover, a relatively high proportion of patients had complex comorbidities such as immunodeficiencies and metabolic disorders, which could affect the risk–benefit profile of HFOV and contribute to outcome heterogeneity across populations. Third, although patients who received HFOV for less than 24 h were excluded to reduce exposure heterogeneity, the actual duration of HFOV use among included patients varied and was not further analyzed, which may also contribute to residual confounding. Lastly, the relatively small sample size, especially after matching, may have reduced the statistical power to detect differences in secondary outcomes. Importantly, as a retrospective observational study, our findings should be interpreted as associations; genetic matching improves balance of measured covariates but cannot exclude residual and unmeasured confounding, and therefore causal inference is not warranted.

Future research should prioritize large-scale, multicenter observational or retrospective studies to further evaluate the effects of HFOV in different severities of PARDS and its potential for combined use with other therapeutic strategies, such as ECMO. These studies should include more detailed patient stratification analyses, particularly regarding its effects at different P/F levels. Additionally, further investigation is needed to explore the potential benefits of HFOV in specific patient subgroups, especially those with severe hypoxemia who respond poorly to CMV treatment.

## 5 Conclusion

In summary, this study used high standards for case inclusion and rigorous genetic matching to analyze the effectiveness of HFOV in children with moderate to severe PARDS. The findings suggest that early use of HFOV may be associated with higher mortality in this population and, compared to CMV, may particularly reduce the 28-day survival rate in children with moderate PARDS. This study provides important insights into the indications and application strategies of HFOV. Future studies are needed to further explore the role of HFOV in different patient groups and evaluate its combined use with other treatment strategies, aiming to provide more reliable scientific evidence for clinical decision-making.

## Data Availability

The datasets presented in this study can be found in online repositories. The names of the repository/repositories and accession number(s) can be found at: HD (2024), “Early HFOV on Outcomes in Children with Moderate to Severe ARDS”, Mendeley Data, V1, DOI: 10.17632/5pcjfzytgj.1.
