# Supplementary material for: Re-evaluating early high-frequency oscillatory ventilation in moderate-to-severe pediatric ARDS: evidence from a genetic matching analysis
Source: Front Med (Lausanne). 2026 Mar 25;13:1725518. doi: 10.3389/fmed.2026.1725518 (PMC13057540; doi:10.3389/fmed.2026.1725518)
Supplement: Supplementary file 1 [file Table_1.docx]

**Supplementary Table S1. Timing and patterns of HFOV exposure within the first 7 days**

| Variable | Value |
| --- | --- |
| HFOV initiation day within first 7 days | 4 (2–6) |
| Days on CMV within first 7 days before HFOV | 3 (1–5) |
| HFOV duration within first 7 days | 4 (2–6) |
| Initiation timing category: Day 1–2 | 14 (26.4%) |
| Initiation timing category: Day 3–4 | 13 (24.5%) |
| Initiation timing category: Day 5–7 | 26 (49.1%) |
| HFOV days within first 7 days = 1 | 13 (24.5%) |
| HFOV days within first 7 days = 2 | 4 (7.5%) |
| HFOV days within first 7 days = 3 | 9 (17.0%) |
| HFOV days within first 7 days = 4 | 6 (11.3%) |
| HFOV days within first 7 days = 5 | 7 (13.2%) |
| HFOV days within first 7 days = 6 | 8 (15.1%) |
| HFOV days within first 7 days = 7 | 6 (11.3%) |

Note: Values are median (IQR) or n (%). HFOV initiation day was defined as the first day of HFOV use within the first 7 days after PARDS diagnosis based on daily ventilator-mode records; all HFOV patients received CMV before HFOV and no crossover back to CMV occurred within the first 7 days.
